# Evaluation of Anti-SARS-Cov-2 S-RBD IgG Antibodies after COVID-19 mRNA BNT162b2 Vaccine

**DOI:** 10.3390/diagnostics11071135

**Published:** 2021-06-22

**Authors:** Bruna Lo Sasso, Rosaria Vincenza Giglio, Matteo Vidali, Concetta Scazzone, Giulia Bivona, Caterina Maria Gambino, Anna Maria Ciaccio, Luisa Agnello, Marcello Ciaccio

**Affiliations:** 1 Department of Biomedicine, Neurosciences and Advanced Diagnostics, Institute of Clinical Biochemistry, Clinical Molecular Medicine and Laboratory Medicine, University of Palermo, 90127 Palermo, Italy; bruna.losasso@unipa.it (B.L.S.); rosaria.vincenza.giglio@alice.it (R.V.G.); concetta.scazzone@unipa.it (C.S.); giulia.bivona@unipa.it (G.B.); cmgambino@libero.it (C.M.G.); luisa.agnello@unipa.it (L.A.); 2 Department of Laboratory Medicine, University Hospital “P. Giaccone”, 90127 Palermo, Italy; 3 Foundation IRCCS Ca’ Granda Ospedale Maggiore Policlinico, 20121 Milan, Italy; matteo.vidali@gmail.com; 4 Unit of Clinical Biochemistry, University of Palermo, 90127 Palermo, Italy; amciaccio21@gmail.com

**Keywords:** antibodies, COVID-19, immunosurvelliance, SARS-CoV-2, S-RBD IgG, spike, vaccination, vaccine, kinetic

## Abstract

(1) Background: The evaluation of anti-spike protein receptor-binding domain (S-RBD) antibodies represents a useful tool to estimate the individual protection against Severe Acute Respiratory Syndrome CoronaVirus 2 (SARS-CoV-2) infection; (2) Methods: We evaluated anti S-RBD IgG levels by indirect chemiluminescence immunoassay on Maglumi 800 (SNIBE, California) in 2248 vaccinated subjects without previous SARS-CoV-2 infection, 91 vaccinated individuals recovered from COVID-19, and 268 individuals recovered from COVID-19 who had not been vaccinated. Among those who were healthy and vaccinated, 352 subjects performed a re-dosing after about 72 days from the first measurement. (3) Results: Anti S-RBD IgG levels were lower in subjects with previous infection than vaccinated subjects, with or without previous infection (*p* < 0.001). No difference was observed between vaccinated subjects, with and without previous SARS-CoV-2 infection. Overall, anti-RBD IgG levels were higher in females than males (2110 vs. 1341 BAU/mL; *p* < 0.001) as well as in subjects with symptoms after vaccination than asymptomatic ones (2085 vs. 1332 BAU/mL; *p* = 0.001) and lower in older than younger subjects. Finally, a significant decrease in anti-RBD IgG levels was observed within a short period from a complete two-dose cycle vaccination. (4) Conclusions: Our results show an efficacy antibody response after vaccination with age-, time- and sex-related differences.

## 1. Introduction

Severe Acute Respiratory Syndrome CoronaVirus 2 (SARS-CoV-2), causing Coronavirus Disease 2019 (COVID-19), continues to evolve at a rapid pace. From December 2019 to May 2021, over 159,319,384 COVID-19 cases were confirmed, with over 3,311,780 deaths worldwide [1]. Consequently, SARS-CoV-2 infection is endangering the health systems of all countries and the global economy of the world.

The disease’s course is highly unpredictable, ranging from asymptomatic forms to acute respiratory distress syndrome and death [2]. It is noteworthy that SARS-CoV-2 infection could cause long-term sequelae, which are under investigation [3,4,5].

Vaccines against SARS-CoV-2 have been rapidly developed in order to limit the further spread of the virus and curb COVID-19 morbidity and mortality [6,7]. Overall, COVID-19 vaccines, by different mechanisms, induce innate and adaptive immune responses. The latter involves the cellular response (T cells) and the antibody response (B cells), which lead to the production of antibodies directed against different antigens of SARS-CoV-2.

**Figure 1:**
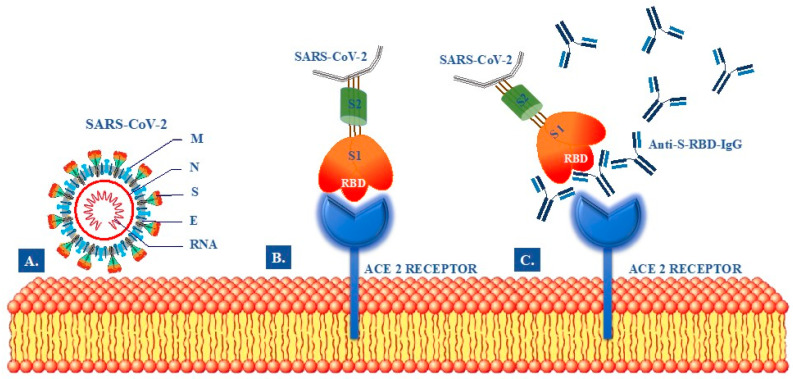
Schematic illustration of postulated mechanism modulating SARS-CoV-2 attachment, fusion and neutralizing activity of anti S-RBD IgG SARS-CoV-2 antibodies. (**A**) Structural components SARS-CoV-2: Envelop Protein (E), Membrane glycoprotein (M), Nucleocapside protein (N), RiboNucleic Acid (RNA), Spike protein (S). (**B**) Binding of Receptor-Binding Domain (RBD) to the Angiotensin-converting enzyme 2 (ACE2) Receptor. (**C**) Inhibition of S-RBD binding to the ACE2 receptor by neutralizing anti-S-RBD IgG antibodies.

It is now well-known that the virus has a pericapsid and four structural proteins: spike protein (S), membrane protein (M), pericapsid protein (E), and nucleocapsid protein (N) (Figure 1A) [8,9,10]. Among the four structural proteins of the SARS-CoV-2, the S and the N are the main immunogens. Protein S has an essential role in viral binding, fusion and replication within the host cell by interacting with the angiotensin-converting enzyme human 2 (ACE2) [8]. The protein S comprises an N-terminal S1 subunit responsible for binding the virus to the receptor and an S2 C-terminal subunit responsible for the fusion of the virus with the cell membrane. S1 is further divided into an N-terminal domain (NTD) and a receptor-binding domain (RBD). The RBD within S1 interacts directly with the receptors of the host cells. Thus, antibodies directed against RBD domain could block the pathogen entry into target cells (Figure 1B).

Recently, several COVID-19 serological diagnostic tests have been developed [11,12]. They are based on the detection of antibodies against all or part of the N or S proteins of SARS-CoV-2. In particular, the commercial tests are usually designed to detect antibodies against SARS-CoV-2 N, S1, S2, S-RBD, or their combinations. It is noteworthy that the evaluation of antibodies against S-RBD IgG is the most important to assess the protection against SARS-CoV-2 infection due to their neutralizing activity (Figure 1C). However, since SARS-CoV-2 is a newly emerging virus, the antibody response in COVID-19 patients and, especially, in vaccinated subjects remains largely unknown.

The aim of this study was to evaluate the anti S-RBD IgG levels on a large cohort consisting of recipients of COVID-19 mRNA BNT162b2 (Pfizer-BioNTech) vaccine, with or without previous SARS-CoV-2 infection, and recovered COVID-19 patients that did not receive the vaccination.

## 2. Materials and Methods

### 2.1. Study Population

This was an observational, single-center study performed at the University Hospital “P. Giaccone” of Palermo, Italy. All consecutive subjects presenting to the Laboratory Medicine Unit to measure anti S-RBD IgG levels, from February to May 2021, were enrolled in the study. We included a total of 2607 subjects stratified into three groups as follows: 2248 vaccinated subjects without previous SARS-CoV-2 infection, 91 vaccinated subjects recovered from COVID-19, and 268 subjects recovered from COVID-19 not vaccinated.

All vaccinated subjects received two doses of Pfizer-BioNTech vaccine (Pfizer Inc., New York, NY; BioNTech SE, Mainz, Germany) 21 days apart. In recovered COVID-19 subjects, the diagnosis of SARS-CoV-2 infection was confirmed by a positive real-time reverse transcription-polymerase chain reaction mainly using naso-oropharyngeal swabs, in accordance with guidelines [13]. All recovered COVID-19 subjects developed asymptomatic or paucisymptomatic forms of the disease, and none were hospitalized for SARS-CoV-2 infection.

In all vaccinated subjects, anti S-RBD IgG levels were measured between the tenth and twentieth days after the second dose of mRNA BNT162b2 vaccine. Additionally, the second measurement of anti S-RBD IgG levels was available in 352 subjects 72 (55–83) days after the first measurement.

Among subjects with the previous infection that did not receive the vaccination, the median (IQR) interval between infection (as assessed by the first positive molecular test) and anti S-RBD IgG levels measurement was 73 (51–101) days.

Demographical and clinical information, including previous infection and history of adverse events to vaccination, were recorded for each subject.

### 2.2. Biochemical Analysis

The serum anti S-RBD IgG levels were measured on fresh samples obtained after centrifugation for 15 min at 4000× *g* at room temperature of whole blood collected in dry tubes. The measurement was performed by indirect chemiluminescence immunoassay on Maglumi 800 (SNIBE—Shenzhen New Industries Biomedical Engineering Co., Ltd, Shenzhen, China) instrumentation, according to the manufacturer’s instructions. The assay has a limit of detection of 0.041 Binding Antibodies Units (BAU)/mL, as declared by the manufacturer. The unit of measurement used is in accordance with the latest notification received from World Health Organization (WHO) (Notice WHO Standard (20/136) Unit Conversion—RN21040201).

### 2.3. Statistical Analysis

Statistical analyses were performed by SPSS statistical software v.17.0 (SPSS Inc., Chicago, IL, USA) and R Language v.4.0.3 (R Foundation for Statistical Computing, Vienna, Austria). Normality distribution was assessed preliminarily by q-q plot, Kolmogorov–Smirnov and Shapiro–Wilk tests. Quantitative variables were expressed by the median and interquartile range (IQR), while categorical variables by absolute and relative frequency. Differences between groups for continuous and categorical variables were estimated, respectively, by non-parametric Kruskal-Wallis test (if >2 groups) or Mann–Whitney U-test (with Bonferroni’s correction when needed) and Chi-squared test. The correlation was evaluated by the non-parametric Spearman test. GLM was used to estimate predictors of anti-RBD IgG response.

## 3. Results

A total of 2607 subjects were included in the study. Demographic variables and anti S-RBD IgG levels are shown in Table 1.

**Figure 2:**
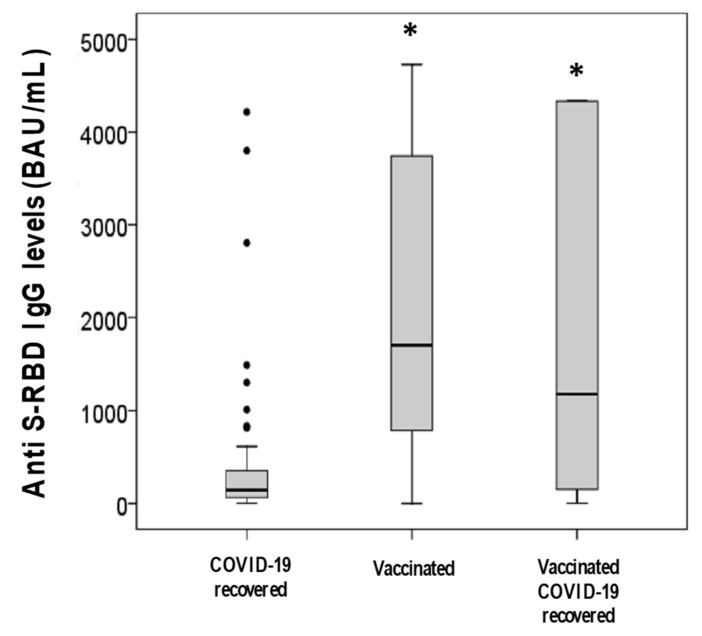
Anti S-RBD IgG levels in the three groups investigated * *p* < 0.001 vs. “COVID-19 recovered” group, Bonferroni’s correction.

Groups were comparable for sex (*p* = 0.226) but differed significantly for age (*p* = 0.043) and anti S-RBD IgG levels (*p* < 0.001). Vaccinated subjects with previous SARS-CoV-2 infection were significantly younger than both vaccinated subjects without previous infection (Bonferroni’s correction, *p* = 0.014) and subjects with the previous infection (*p* = 0.016). Anti S-RBD IgG levels were significantly lower in subjects with the previous infection than vaccinated subjects, both with and without previous infection (Bonferroni’s correction, both *p* < 0.001). No difference was observed between vaccinated subjects with and without previous SARS-CoV-2 infection (*p* = 0.118) (Table 1 and Figure 2).

In the vaccinated group, with or without previous infection, the information on the development or otherwise of adverse events to vaccination was available in 54 (59%) and 1933 (86%) subjects, respectively. A total of 1148 subjects displayed one or more symptoms after vaccination (Table 2). After the first dose, the adverse effect with the highest prevalence was local or diffuse muscular pain (16.8%), with a minority of subjects displaying only systemic effects or in association with other symptoms (Table 2). After the second dose, 21.8% of subjects displayed only local effects, whereas 15.8% of subjects developed systemic effects only, and 13.9% of subjects developed systemic effects together with one or more additional symptoms (Table 2).

**Figure 3:**
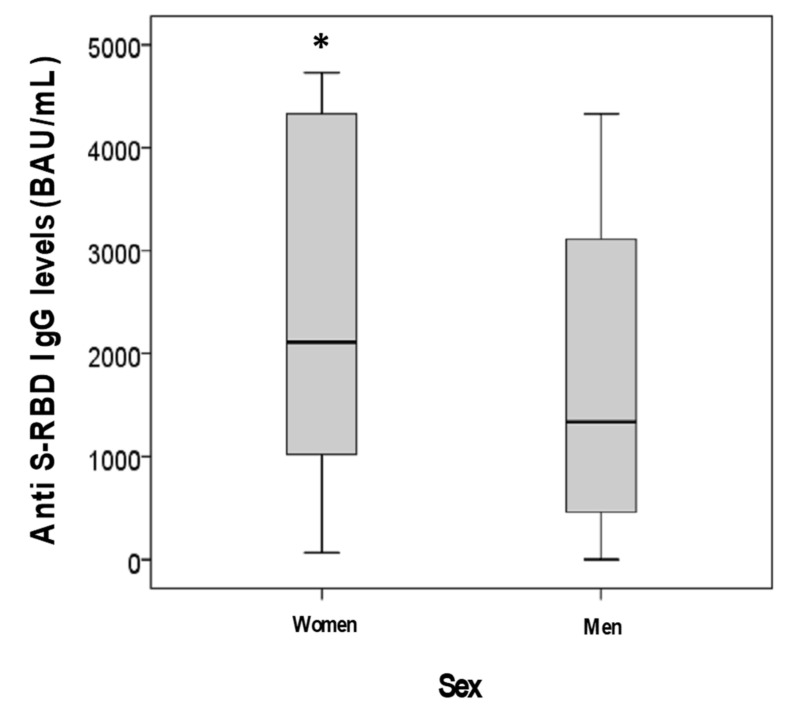
Anti S-RBD IgG levels in subjects sub-grouped according to sex (* *p* < 0.001 vs. men).

**Figure 4:**
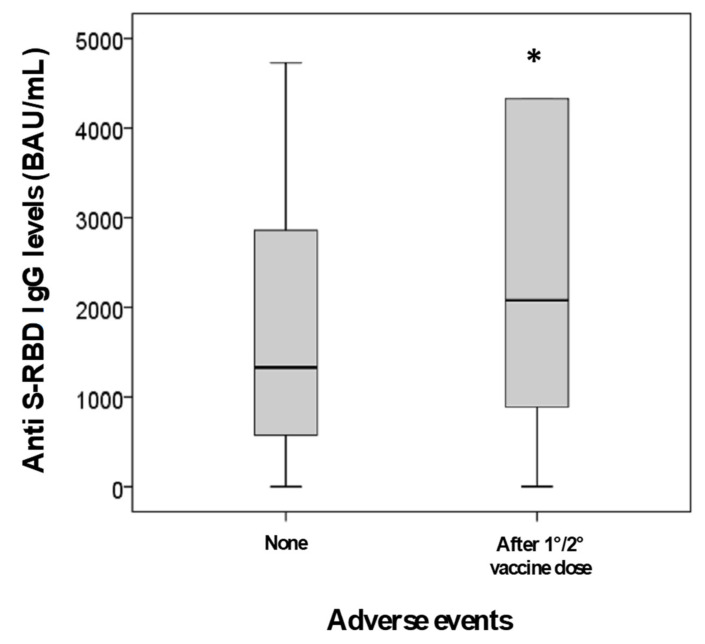
Anti S-RBD IgG levels in subjects sub-grouped by the absence or the presence of symptoms after first and/or second dose (* *p* = 0.001 vs. absence of symptoms).

**Figure 5:**
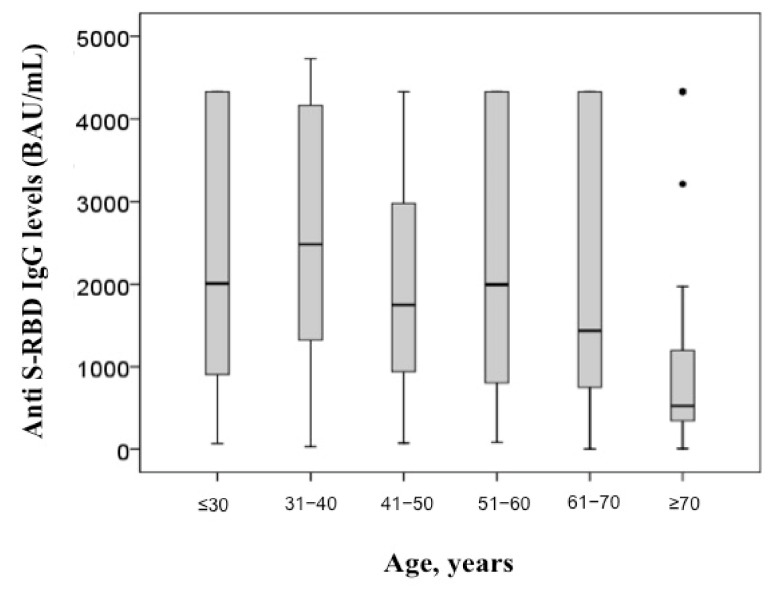
Anti S-RBD IgG levels in subjects sub-grouped by age.

A model for anti S-RBD IgG response was further evaluated in 2112 vaccinated subjects, including both those with and without previous infection, for whom we had complete data collection, using as predictors age, sex, previous infection and the development of one or more symptoms after vaccination (after first and/or second administration). During GLM analysis, age (*p* = 0.012), the development of adverse effects after vaccination (*p* = 0.001) and sex (*p* = 0.003), but not the presence of previous infection (*p* = 0.660), were shown to be independent predictors of anti S-RBD IgG levels. In these 2112 subjects, anti S-RBD IgG levels were, indeed, significantly higher in women than men (2110 vs. 1341 BAU/mL; *p* < 0.001) (Figure 3) as well as in subjects with one or more symptoms after vaccination than asymptomatic ones (2085 vs. 1332 BAU/mL; *p* = 0.001) (Figure 4). Additionally, anti S-RBD IgG levels decreased with age (rho = −0.190; *p* < 0.001) (Figure 5).

Subjects with anti-RBD IgG response tested at least two different time points after a complete two-dose cycle of vaccination were further evaluated. Change in anti S-RBD IgG response per day was calculated as [(RBDt1 − RBDt2)/RBDt1]/(t2 − t1), where RBDt1 and RBDt2 indicate anti S-RBD IgG levels at the two different time points, while t2 − t1 indicates time interval in days between blood sampling.

**Figure 6:**
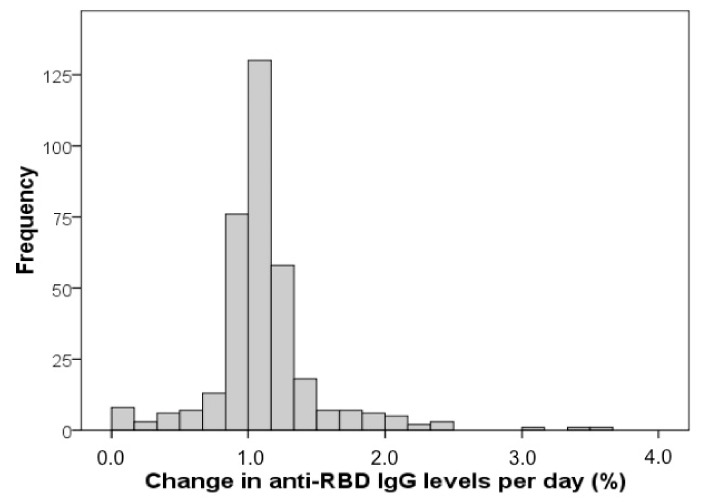
Distribution shown by histogram of the change (%) per day of anti S-RBD IgG levels. X axis represents % change in anti S-RBD IG levels per day, while y axis represents the absolute frequency of patients.

Data were available for 352 subjects (M 45%, F 55%), median (IQR) age 52 (36–61) years. The median (IQR) anti S-RBD IgG response after both doses was 1372 (523–2610) BAU/mL. The median (IQR) time interval between blood sampling time points after vaccination (both doses) was 72 (55–83) days. Median (IQR) change in anti S-RBD IgG response per day was 0.011 (0.010–0.012), indicating a median −1.1% decrease in anti S-RBD IgG levels per day with respect to the basal value (Figure 6). Figure 6 clearly shows that the majority of patients had a median change of IgG levels of −1.1% per day. Females displayed significantly higher basal anti S-RBD IgG levels (median 1537 vs. 1122 BAU/mL; *p* = 0.003) than males; however, no significant difference was found in change in anti S-RBD IgG response (median 0.011 vs. 0.011; *p* = 0.810). Although a weak significant inverse correlation was found between basal anti S-RBD IgG levels and age (rho = −0.140; *p* = 0.009), no association was found between age and change in anti S-RBD IgG response (rho = 0.041; *p* = 0.441).

## 4. Discussion

The SARS-CoV-2 infection represents one of the most serious global health threats due to its severity and rapid spread worldwide. Thus, vaccines have been rapidly developed in order to control SARS-CoV-2 diffusion among the population. The immune surveillance by the measurement of antigen-specific antibody levels is fundamental to assess vaccine efficacy. Although SARS-CoV-2 infection can induce the production of antibodies recognizing different viral antigens, antibodies directed against the RBD are the most relevant due to their neutralizing activity [6]. Accordingly, most of the SARS-CoV-2 vaccines have been developed to induce the production of antibodies against the SARS-CoV-2 spike protein. Therefore, the measurement of circulating anti S-RBD IgG levels may provide precious information on acquired immunity against SARS-CoV-2.

In this large observational study, we sought to evaluate the anti S-RBD IgG levels following the natural infection, the vaccination, and the vaccination after SARS-CoV-2 infection recovers. The main findings of our study can be summarised as follows: (i) anti S-RBD IgG levels were significantly lower in recovered COVID-19 subjects than vaccinated subjects, both with or without previous SARS-CoV-2 infection; (ii) among all vaccinated subjects, age, adverse effects to vaccination, and sex were independent predictors of anti S-RBD IgG levels. Specifically, females and subjects developing adverse effects showed the highest levels of antibodies, while older subjects the lowest; (iii) finally, we found a significant decrease in anti S-RBD IgG levels within a short period. Interestingly, the decrease rate was not influenced by age and sex.

Our findings are in accordance with Muller et al., who measured SARS-CoV-2 spike S1 IgG titers by enzyme-linked immunosorbent assay in a small cohort, consisting of 176 recipients of the mRNA BNT162b2 COVID-19 vaccine [13]. They showed that the elderly had significantly lower levels of antibodies than young subjects.

Two studies evaluated the total levels of anti-SARS-CoV-2 RBD antibodies, including both IgM and IgG, after vaccine administration [14,15]. Salvagno et al. showed that age and sex were independent predictors of antibody levels [14]. Specifically, they found an inverse association between total anti-SARS-CoV-2 RBD antibody levels and age as well as male sex. Similarly, Terpos et al. found that females had higher levels of total anti-SARS-CoV-2 RBD antibodies than males in octogenarian vaccine recipients [15]. In our study, we evaluated IgG anti S-RBD by an automatic platform based on chemiluminescence. The evaluation of IgG is more appropriate than the total antibodies levels for the assessment of the immunosurveillance post-vaccine. Indeed, IgMs are produced early after infection and later replaced with IgGs, which represent reliable indicators of long-term immunity.

To the best of our knowledge, this is the largest sample size study performed on the Italian population to date. Our study provides important information on the kinetics of antibodies, both in terms of time and titers. Although most of the recipients had detectable antibody levels 72 (55–83) days after the first measurement, we observed a significant antibody decay rate over time. This is an important issue that must be further investigated in order to better plan the individual vaccine programme.

Overall, precious information on vaccine efficacy is emerging. Specifically, demographical variants have been identified as an important predictor of the humoral response. Both our own and previous findings suggest that age and sex significantly influence vaccine-induced immunogenicity. Specifically, males and those of an old age seem to be associated with a less efficacy humoral response. Thus, it would be pivotal to understand if such categories are more susceptible to SARS-CoV-2 infection and to develop more aggressive forms of COVID-19. Accordingly, it is plausible that men and the elderly should be strictly monitored and may require earlier revaccination or/and an increased vaccine dose to ensure stronger, long-lasting immunity and protection against infection. We also found that the antibody titer decreases significantly within a short period after the 2nd dose of vaccine administration. However, since the COVID-19 vaccines have been developed recently, other important questions must be addressed, including the durability of protection over a long period after vaccination and the determination of the effect of a booster dose to extend the duration of immunity against SARS-CoV-2 infection.

The present study has some limitations. First, we included a low number of both vaccinated recovered COVID-19 subjects and no vaccinated recovered COVID-19 subjects. Furthermore, the recovered COVID-19 subjects were not hospitalized during their SARS-CoV-2 infection. Finally, the study is not controlled, and we did not measure the IgG levels in vaccinated COVID-19 recovers before vaccination.

## 5. Conclusions

An extensive vaccination campaign is underway with a total of 1,545,967,545 vaccine doses administered and the number is growing daily [1]. Evaluating the humoral response to the vaccine is a hot topic worldwide in order to unravel the efficacy of the vaccine and, consequently, to tailor the optimal vaccination strategy. This large observational study showed a robust antibody response after mRNA BNT162b2 COVID-19 vaccine administration, characterized by a good antibody production with age- and sex-related differences. Additionally, we showed a rapid antibody decay rate within a short period after a completed two-dose vaccine cycle.

## Figures and Tables

**Table 1:** Demographic characteristics and anti S-RBD IgG levels in the three groups was investigated.

	Vaccinated (*n* = 2248)	Vaccinated COVID-19 Recovered (*n* = 91)	COVID-19 Recovered (*n* = 268)	*p*
Age, years (median, IQR)	57 (41–65)	51 (36–56)	56 (47–63)	0.043
Sex, M *n* (%)	1169 (52)	37 (41)	158 (59)	0.226
Anti S-RBD IgG, BAU/mL (median, IQR)	1703 (786–3778)	1175 (146–4330)	142 (60–366)	<0.001

**Table 2:** Adverse events after first or second dose of vaccine.

Adverse Events	After First Dose	After Second Dose
Local and/or muscle pain	16.8%	21.8%
Only respiratory effects	0.2%	0.5%
Only systemic effects	4.6%	15.8%
Systemic + local and/or muscle/arm pain and/or respiratory effects	4.1%	13.9%
Severe systemic reaction	0.2%	0%
No reaction	74.1%	48.0%

## Data Availability

Derived data supporting the findings of this study are available from the corresponding author on request.
